# Cellvibrio chitinivorans sp. nov., a chitinolytic bacterium isolated from an intertidal mudflat

**DOI:** 10.1099/ijsem.0.006827

**Published:** 2025-06-27

**Authors:** Yanzhe Xin, Xing Qin, Honghai Zhang, Tao Dong, Xiaolu Wang, Yaru Wang, Yuan Wang, Tao Tu, Huoqing Huang, Bin Yao, Huiying Luo

**Affiliations:** 1 State Key Laboratory of Animal Nutrition and Feeding, Institute of Animal Sciences, Chinese Academy of Agricultural Sciences, Beijing 10093, PR China

**Keywords:** *Cellvibrio chitinivorans*, chitinolytic bacterium, intertidal mudflat, new taxa

## Abstract

A novel chitinolytic bacterium, designated as NN19^T^, was isolated from the intertidal mudflat in Leizhou, Guangdong Province, PR China, and subjected to polyphasic taxonomy studies. Strain NN19^T^ is a Gram-negative short rod-shaped bacterium that is motile via a single polar flagellum. Growth occurred at temperatures of 15 °C and 37 °C (optimal 30 °C), pH 6.5–8.0 (optimal pH 7.0) and sodium chloride concentration of 0%–3% (optimal 0.5%–1%). It was identified as a *Cellvibrio* species based on the results of 16S rRNA gene sequence analysis, and it showed the highest degree of sequence similarity with *Cellvibrio fontiphilus* MVW-40^T^ (97.87%). The main fatty acids of NN19^T^ were found to be summed feature 3 (C_16 : 1_* ω*7*c*/C_16 : 1_* ω*6*c*), C_16 : 0_ and C_18 : 1_* ω*7*c*. The polar lipid profile included diphosphatidylglycerol, phosphatidylethanolamine, phosphatidylglycerol and phospholipid. The respiratory quinone was shown to be ubiquinone-8. Strain NN19^T^ had a genome of 4.46 Mb with a G+C content of 47.82%. The average nucleotide identity and digital DNA–DNA hybridization value between strain NN19^T^ and *C. fontiphilus* MVW-40^T^ are 75.47% and 20.4%, respectively. Based on phylogenetic analysis and phenotypic data, we concluded that strain NN19^T^ represents a novel species belonging to the genus *Cellvibrio*, for which the name *Cellvibrio chitinivorans* sp. nov. is proposed. The type strain is NN19^T^ (=MCCC 1K08847^T^=KCTC 8393^T^).

## Introduction

The genus *Cellvibrio* was originally proposed by Winogradsky in 1929 with two species: *Cellvibrio ochraceus* and *Cellvibrio flavescens* [1]. As of July 2024 (during the preparation of this manuscript), the genus *Cellvibrio* comprises 11 validly published species listed in the List of Prokaryotic names with Standing in Nomenclature [2]. Members of this genus have been frequently isolated from soil, rhizosphere or aquatic environments [3,5]. *Cellvibrio* species are Gram-negative, aerobic bacteria with rod-shaped cells with polar flagella [6]. The predominant fatty acids of this genus are unsaturated summed feature 3 (C_16 : 1_* ω*7*c*/C_16 : 1_* ω*6*c*) and C_18 : 1_* ω*7*c*, along with saturated C_16 : 0_, and the DNA has a G+C content of 44.2 to 53.3% [5,6]. The genus has been reported to have extensive hydrolytic ability against complex polysaccharides [3,7], especially *Cellvibrio japonicus* [8,9]. The hydrolytic enzymes of *C. japonicus*, including cellulase, hemicellulose, pectinase and chitinase, have been studied in detail both biochemically and structurally [8]. It is important to unravel the complex mechanisms of lignocellulose and chitin degradation in the environment, as these enzymes hold great potential for industrial applications.

Chitin, primarily found in the exoskeletons of crustaceans such as shrimp and crabs, is a key structural component of protective or supportive extracellular matrices [10]. Intertidal sediments, serving as typical habitats for crustaceans (e.g. ghost crabs and clams), harbour specialized microbial communities with chitinolytic potential [11]. In this study, we isolated a highly efficient chitin-degrading strain, NN19^T^, from intertidal sediment samples using a combination of chitin-enriched cultivation and enzyme activity-based screening strategies. Phylogenetic analysis revealed its close affiliation with members of the genus *Cellvibrio*. We employed a polyphasic taxonomic approach to comprehensively evaluate the taxonomic status of this putative novel species within the genus *Cellvibrio*.

## Sample collection and isolation

**Fig. 1.**
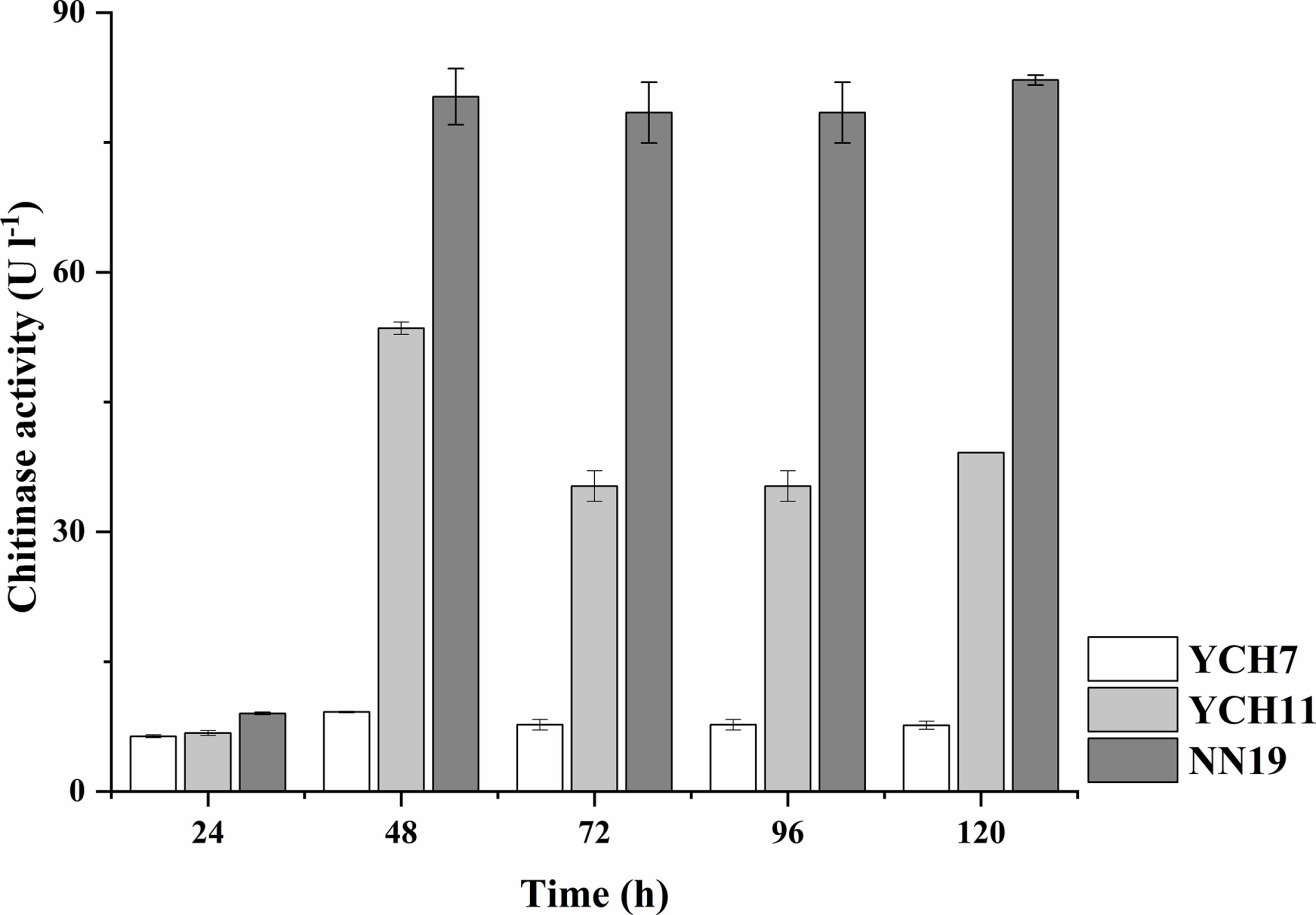
Alterations in the chitinase activity within the culture supernatant.

Strain NN19^T^ was isolated from the intertidal mudflat in Leizhou City, Guangdong Province, PR China (109° 56′ 37″ N 20° 28′ 44″ E). This bacterium can grow on a medium with chitin as the sole carbon source. Colloidal chitin was prepared according to the method of Hua *et al*. [12] with slight modifications. Briefly, 4 g of chitin powder from shrimp shells was completely dissolved in 40 ml of concentrated hydrochloric acid and left to stand for 50 min. The solution was mixed with 1 l of distilled water precooled to 4 °C and left to stand overnight at 4 °C. The precipitate collected by centrifugation was washed with distilled water until the pH reached 7.0. Then, the colloidal chitin was adjusted to 100 ml with distilled water and stored at 4 °C. The samples were suspended in 1% w/v colloidal chitin liquid medium consisting of 1.0 g l^−1^ KNO_3_, 0.5 g l^−1^ K_2_HPO_4_, 0.5 g l^−1^ MgSO_4_·7H_2_O, 0.5 g l^−1^ NaCl and 0.01 g l^−1^ FeSO_4_·7H_2_O and incubated at 30 °C with shaking at 180 r.p.m for 5 days. The enriched solution was diluted 10^6^-fold with 0.85% physiological saline and applied to agar plates containing 1% colloidal chitin solid medium prepared by adding 2% agar to the 1% colloidal chitin liquid medium and cultured at 30 °C for 5 days. Individual colonies on the plates were purified on the same medium. Three pure bacterial strains (designated YCH7, YCH11 and NN19^T^) were ultimately obtained, demonstrating robust growth on chitin as the sole carbon source. These strains were inoculated into liquid medium containing 1% (w/v) colloidal chitin and incubated under shaking conditions (150 r.p.m.) at 30 °C for 5 days. Chitinase activity in the culture supernatant was quantified every 24 h using the 3,5-dinitrosalicylic acid method [13], with strains exhibiting the highest enzymatic activity prioritized as superior chitin degraders. One unit of chitinase activity (U) was defined as the amount of enzyme required to release 1 µmol of reducing sugar (expressed as *N*-acetylglucosamine equivalents) per minute under standardized assay conditions. Strain NN19^T^ exhibited the highest chitinase activity (80 U l^−1^ at 48 h), as shown in Fig. 1, and was therefore selected as the most promising candidate.

## 16S rRNA gene phylogeny

**Fig. 2.**
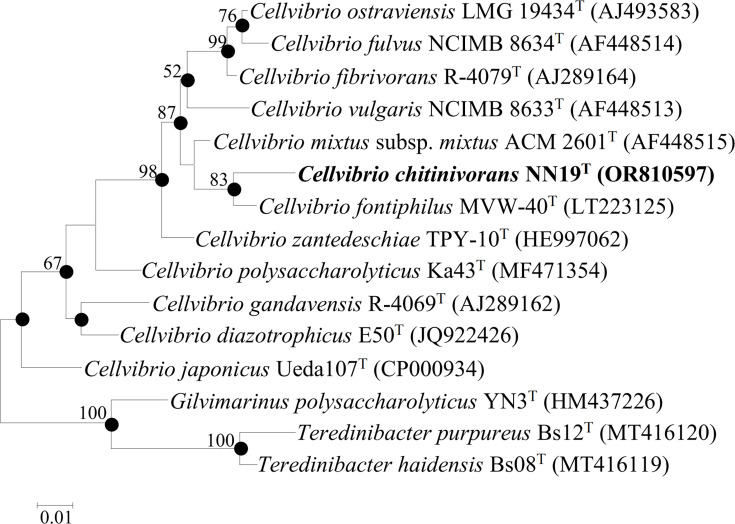
Maximum-likelihood tree showing the phylogenetic positions of strain NN19^T^ and related taxa based on 16S rRNA gene sequences. Bootstrap values (expressed as percentages of 1,000 replicates) are shown at branch points. Bar: 0.01 substitutions per nucleotide position.

Genomic DNA of NN19^T^ was prepared using a Bacterial Genomic DNA Extraction Kit (Vazyme, Nanjing, China). The 16S rRNA gene was amplified by PCR using the universal primers 27F and 1492R [14]. Bidirectional sequencing (forward and reverse strands) was performed by Beijing Liuhe Huada Gene Technology Co., Ltd., and consensus sequences were generated to ensure accuracy. Sequence comparisons were performed using the EzBioCloud database (www.ezbiocloud.net/identify) and mega X [15]. A phylogenetic tree was constructed based on the Kimura two-parameter model for distance estimation using the maximum-likelihood, neighbour-joining and minimum evolution methods with 1,000 bootstrap replicates [16,18]. Sequence identity analysis showed that the strain NN19^T^ (GenBank accession number: OR810597) had the highest 16S rRNA sequence identity with *Cellvibrio fontiphilus* MVW-40^T^ (97.87%) [4], followed by *Cellvibrio fibrivorans* R-4079^T^ (97.44%) [19], *Cellvibrio mixtus* subsp. *mixtus* ACM 2601^T^ (97.11%) [20], *Cellvibrio ostraviensis* LMG 19434^T^ (97.08%) [19] and *Cellvibrio fulvus* NCIMB 8634^T^ (96.7%) [6]. On the phylogenetic trees [Figs 2 and S3 (available in the online Supplementary Material) and S4], strain NN19^T^ formed a branch with *C. fontiphilus* MVW-40^T^, suggesting that it could belong to the genus *Cellvibrio*.

## Genomic feature analysis

**Fig. 3.**
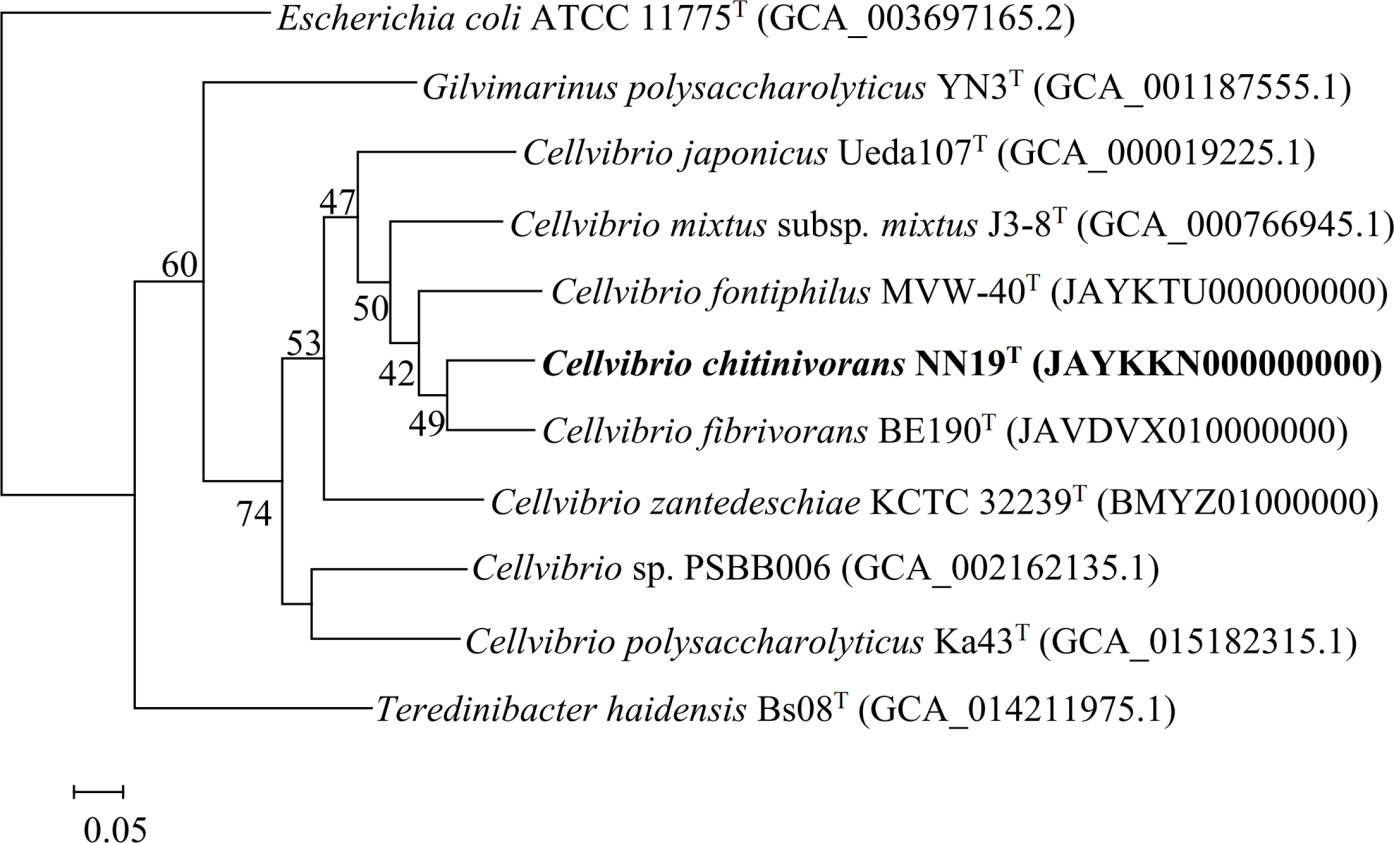
Phylogenetic tree of the genome between strain NN19^T^ and closely related strains of the *Cellvibrio* genus.

The genome of NN19^T^ was sequenced by Allwegene Technology (Beijing, China). The company used the Illumina NovaSeq PE150 sequencing platform (Illumina, San Diego, CA, USA). To ensure data reliability, raw sequencing reads underwent stringent filtering to remove low-quality bases (Q≤20), ambiguous nucleotides (*N*>10%) and adapter contamination (overlap ≥15 bp with ≤3 mismatches). We generated 1.17 Gbp of clean data with a coverage depth of 227-fold. Clean data were assembled using SOAPdenovo2 (version 2.04) with multiple k-mer parameters, SPAdes (version 3.15.5) and Abyss (version 2.3.7), employing optimized parameters to generate minimal scaffolds. The assembly results from these tools were integrated via CISA (version 1.3) to select consensus-optimized scaffolds, followed by gap filling using GapCloser (version 1.2). Low-coverage reads (<0.35× the average depth) and fragments <500 bp were filtered out to obtain the final assembled sequence for downstream gene prediction. The quality and completeness of the genome assembly of NN19^T^ were evaluated using CheckM (http://ecogenomics.github.io/CheckM/) [21]. The genome assembly statistics and completeness analysis of strain NN19^T^ are detailed in Tables S5 and S6, respectively. The CheckM results showed that the genome completeness was 100%, with a contamination level of 0.24%. The genome accession number of strain NN19^T^ is JAYKKN000000000, with N50 and L50 values of 762,285 and 2, respectively. The genome size is 4.46 Mb with a G+C content of 47.82% and contains 42 tRNA genes, 3 5S rRNAs, 1 16S rRNA and 1 23S rRNA. The genome phylogenetic tree of NN19^T^ and other closely related strains of the *Cellvibrio* genus was constructed using an up-to-date bacterial core gene set consisting of 92 genes [22]. The results showed that NN19^T^ and *C. fibrivorans* BE190^T^ are in the same branch (Fig. 3). The average nucleotide identity (ANI) and digital DNA–DNA hybridization (dDDH) values between NN19^T^ and *C. fontiphilus* KCTC 52237^T^ were calculated using the OrthoANI (http://www.ezbiocloud.net/tools/ani) algorithm [23] and the genome-to-genome distance calculator (GGDC3.0) [24]. As shown in Table 1, the ANI and dDDH values between NN19^T^ and type strains of closely related *Cellvibrio* species were lower than the standard ANI criterion for species identity (95%–96%) [25] and the standard dDDH cut-off value (70%), respectively [26]. To delve into the chitin degradation mechanism of the NN19^T^ strain, we conducted an exhaustive analysis of the chitin degradation-related proteins within its genome and annotated the genome utilizing the Rapid Annotation Subsystem Technology (RAST) server (https://rast.nmpdr.org/). Currently, the only reported chitin-degrading strain within the genus *Cellvibrio* is *C. japonicus*, which has five annotated chitinase genes and two *β*-*N*-acetylglucosaminidase genes in its genome [27,28]. However, our research results indicate that NN19^T^ predicts the involvement of six chitinases and four *β*-*N*-acetylglucosaminidases in chitin degradation, surpassing the number of chitinolytic enzyme genes found in *C. japonicus*. Additionally, a comparative genomic analysis between NN19^T^ and other closely related strains is presented in Table S1 for further insights. RAST analysis showed that NN19^T^ had more genes annotated in the 'Protein Metabolism', 'Nucleosides and Nucleotides' and 'Motility and Chemotaxis' subsystem categories than the other three type strains.

**Table 1.** ANI and dDDH values amongst strain NN19^T^ and type strains of closely related *Cellvibrio* species

Strain	ANI (%)	dDDH (%)
*C*. *fontiphilus* KCTC 52237^T^	75.5	20.4
*C*. *mixtus* subsp. *mixtus* J3-8^T^	75.3	20.1
*C*. *japonicus* Ueda107^T^	72.7	19.4
*C*. *fibrivorans* BE190^T^	78.1	21.2
*C*. *zantedeschiae* KCTC 32239^T^	72.5	20.4
*C*. *polysaccharolyticus* Ka43^T^	71.0	20.5

The secondary metabolite-associated biosynthetic gene clusters (BGCs) of strain NN19^T^ and its closely related strains were identified and annotated using antiSMASH version 8.0.0 [29]. Strain NN19^T^ harbours five BGCs, whilst *C. fontiphilus* KCTC 52237^T^, *C. mixtus* subsp. *mixtus* J3-8^T^ and *C. fibrivorans* BE90^T^ contain eight, five and seven BGCs, respectively (Table S2). All strains shared the capacity to synthesize terpene precursor and arylpolyene, whereas NN19^T^ uniquely possesses the acyl_amino_acids BGC, encoding enzymes for amino acid surfactants – a major class of bio-based ingredients in personal care applications [30]. Based on the above analysis, strain NN19^T^ represents a new species of the *Cellvibrio* genus.

## Physiology and chemotaxonomy

NN19^T^ was cultured on trypsin soy agar (TSA) at 30 °C and pH 7.0. The cellular morphology of NN19^T^ was examined by transmission electron microscopy. Gram staining was performed using a commercial kit (Solarbio, Beijing, China). The movement of cells was examined using the hanging drop method [31]. Oxidase activity was assessed using 1% (w/v) *N*,*N*,*N*′,*N*′-tetramethyl-1,4-phenylenediamine. Catalase activity was determined by monitoring the generation of bubbles in a 10% (v/v) hydrogen peroxide solution. The pH range for growth of NN19^T^ was determined in modified R2B medium across pH 3.0–10.0 at 1 pH unit intervals, using the following buffer systems: citrate/phosphate buffer (pH 3.0–7.0), Tris/HCl buffer (pH 8.0–9.0) and sodium carbonate/sodium bicarbonate buffer (pH 10.0) [32]. The growth of NN19^T^ was assessed at different temperatures (4, 10, 15, 20, 25, 30, 37, 41 and 45 °C), and its NaCl tolerance was investigated in modified R2B medium supplemented with NaCl concentrations of 0, 0.5, 1, 2, 3, 4, 5, 6, 7, 8, 9, 10, 12, 15 and 18% (w/v). The strain was Gram-negative with light yellow, slightly sticky, round, short rod-shaped cells that moved via a unipolar flagellum. The average cell diameter and length were 0.5 and 1.2–1.4 µm, respectively (Fig. S1). The strain showed optimal growth at a temperature of 30 °C, pH 6.5–8.0 and NaCl concentration of 0%–3%, with an optimal pH of 7.0 and optimal NaCl concentration of 0.5%–1%. It was positive for oxidase and catalase. The physiological characteristics of two related strains, *C. fontiphilus* KCTC 52237^T^ and *C. mixtus* subsp. *mixtus* LMG 21544^T^, were compared and analysed. The assimilation, enzyme activity and carbon source utilization of NN19^T^ with different substrates were investigated using API 20NE, API ZYM strips (bioMérieux, Marcy-l'Étoile, France) and Biolog GEN III MicroPlate (Biolog, Hayward, CA, USA). The phenotypic characteristics that distinguish strain NN19^T^ from its related species are shown in Table 2. Furthermore, comprehensive Biolog metabolic profiling and ZYM enzyme activity assays revealed that strain NN19^T^ exhibited *β*-glucosidase activity and the ability to metabolize cellobiose, suggesting their cellulose hydrolytic capacity. This aligns with the characteristic cellulose-degrading capability of the genus *Cellvibrio* (Tables S3 and S4). Additionally, NN19^T^ and reference strains were inoculated into a 1% colloidal chitin liquid medium and cultured for 48 h. As shown in Fig. S6, NN19^T^ exhibited the highest degradation capability compared to the control.

**Table 2.** Differential characteristics of strain NN19^T^ and type strains of closely related *Cellvibrio* species

Characteristic	1	2	3	4	5	6	7
Temperature range (optimum) for growth (°C)	15–37 (30)	15–40 (20–30)	10–37 (20–30)	15–30 (25–30)	10–30 (20–30)	15–35 (30)	10–30 (25)
Growth with NaCl (optimum) (%)	0–3 (0.5–1)	0–2 (0)	0–4 (0–2)	0–2 (0.5–1)	0–2 (0–1)	0.5–5.0 (1.0)	0.5–1.0 (1.0)
**API 20NE assay**							
Nitrate reduction	+	+	−	+	−	−	−
Maltose	+	w	+	+	+	−	+
Gluconate	w	−	w	−	−	−	−
*N-*­Acetylglucosamine	+	+	+	+	+	+	−
**API ZYM assay**							
Esterase (C4)	+	+	+	+	+	−	−
Acid phosphatase	+	w	w	+	−	+	−
*α*-Galactosidase	w	+	+	+	+	−	−
*β*-Galactosidase	−	w	w	−	−	+	−
*β*-Glucuronidase	−	−	w	+	+	−	−
*α*-Glucosidase	−	+	+	+	+	−	−
*β*-Glucosidase	w	+	+	+	+	−	−
**Biolog GEN III assay**							
d-Fructose	−	+	−	−	−	−	+
Pectin	−	+	+	nd	+	+	+
l-Malic acid	−	+	−	nd	nd	nd	nd

Strains: 1, NN19T (data from this study); 2, *C. fontiphilus* KCTC 52237T (data from this study); 3, *C. mixtus* subsp. *mixtus* LMG 21544T (data from this study); 4, *C. fibrivorans* LMG 18561T (data from [4]); 5, *C. fulvus* LMG 2847T (data from [4]); 6, *C. diazotrophicus* LMG 27267T (data from [3]); 7, *C. gandavensis* LMG 18551T (data from [3]). +, positive; −, negative; w, weakly positive; nd, not determined.

The fatty acid profile was examined based on a previous study [33]. Briefly, after culturing NN19^T^ cells on TSA medium at 30 °C for 48 h, the fatty acids in the whole cells were extracted according to the standard Sherlock Microbial Identification System (MIS) version 6.0B (MIDI, Inc., Newark, DE, USA). Subsequently, the extracted fatty acids were analysed in detail by GC (Agilent Technologies 6850; Agilent Technologies, Santa Clara, CA, USA), and the identification of fatty acids was completed using the TSBA6.0 database of MIS [34]. The fatty acid compositions of strain NN19^T^ and its closely related species were similar [3,4, 6, 35], with the main fatty acids being summed feature 3 (C_16 : 1_* ω*7*c*/C_16 : 1_* ω*6*c*), C_16 : 0_ and C_18 : 1_* ω*7*c*. However, there were also subtle differences between species (Table 3).

**Table 3.** Cellular fatty acid compositions of strain NN19^T^ and type strains of closely related *Cellvibrio* species

Fatty acid	1	2	3	4	5	6	7
**Straight chain**							
C_10 : 0_	1.7	3.8	3.1	4.2	2.8	2.7	1.3
C_12 : 0_	7.6	4.2	1.1	3.2	7.8	4.8	4.5
C_16 : 0_	17.6	24.6	12.8	24.0	17.4	16.9	22.9
C_17 : 0_	5.0	3.4	10.5	1.1	2.1	2.0	1.6
C_18 : 0_	8.3	2.2	1.5	1.3	4.0	3.8	1.0
**Hydroxy**							
C_10 : 0_ 3-OH	6.6	5.8	8.1	6.4	3.7	3.5	2.1
**Unsaturated**							
C_18 : 1_* ω*7*c*	11.3	10.1	19.8	10.2	12.0	16.4	20.4
Summed feature 3 (C_16 : 1_* ω*7*c*/C_16 : 1_* ω*6*c*)	27.9	34.2	23.2	36.2	27.2	37.7	37.7

Strains: 1, NN19T (data from this study); 2, *C. fontiphilus* KCTC 52237T (data from this study); 3, *C. mixtus* subsp. *mixtus* LMG 21544T (data from this study); 4, *C. fibrivorans* LMG 18561T (data from [4]); 5, *C. fulvus* LMG 2847T (data from [4]); 6, *C. diazotrophicus* LMG 27267T (data from [3]); 7, *C. gandavensis* LMG 18551T (data from [3]). Values are percentages of total fatty acids.

Quinones were extracted and separated into distinct categories using silica gel TLC. Subsequently, these isolated quinones were subjected to further precise analysis by HPLC [36,37]. The respiratory quinone of strain NN19^T^ was ubiquinone-8 (Q-8).

The extraction of polar lipids was conducted as described previously [38], followed by analysis using one-dimensional and two-dimensional TLC [33]. The results of polar lipid analysis showed that NN19^T^ contained diphosphatidylglycerol (DPG), phosphatidylethanolamine (PE), phosphatidylglycerol (PG) and phospholipid (PL). The latter three were also detected in its close relative, * C. fontiphilus* KCTC 52237^T^, but DPG was unique to NN19 (Fig. S2).

## Taxonomic conclusion

Strain NN19^T^ represents a novel species within the genus *Cellvibrio*, as evidenced by 16S rRNA gene sequence identity, ANI and dDDH values. Genomic analysis further revealed the presence of a unique acyl_amino_acid biosynthesis pathway absent in closely related strains of the genus. Phenotypic and biochemical characterization demonstrated both similarities and distinct differences between NN19^T^ and other members of the genus *Cellvibrio*. Notably, NN19^T^ is capable of metabolizing gluconate but lacks the ability to degrade pectin, and it exhibits no detectable *β*-galactosidase or *α*-glucosidase activity. Whilst its fatty acid composition and polar lipid profile align with those of established *Cellvibrio* type strains, subtle distinctions in lipid patterns were observed. In conclusion, phylogenetic, genomic, chemotaxonomic and phenotypic data collectively support the classification of strain NN19^T^ as a novel species within the genus *Cellvibrio*, for which the name *Cellvibrio chitinivorans* sp. nov. is proposed.

## Description of *Cellvibrio chitinivorans* sp. nov.

*Cellvibrio chitinivorans* (chi.ti.ni.vo'rans. N.L. neut. n. *chitinum*, chitin; L. pres. part. *vorans*, devouring; N.L. part. adj. *chitinivorans*, chitin-devouring).

Short Gram-negative rod-shaped cells move via a single polar flagellum. After incubation on TSA medium at 30 °C for 48 h, the colonies were light yellow in colour, slightly sticky and round, with an average cell size of 0.5 µm in diameter and 1.2–1.4 µm in length. The strain grew at 15 °C–37 °C (optimum, 30 °C), pH 6.5–8.0 (optimum, pH 7.0) and 0%–3% NaCl (optimum, 0.5%–1%). Cells were positive for oxidase and catalase. The API 20NE results were positive for nitrate reduction, aesculin hydrolysis, *para*-nitrophenyl-*β*-galactosidase activity and assimilation of glucose, *N*-acetylglucosamine, arabinose, mannose and maltose. The API ZYM results were also positive for alkaline phosphatase, esterase (C4), esterase lipase (C8), leucine arylamidase, valine arylamidase, acid phosphatase and *N*-acetyl-*β*-glucosaminidase. It was also weakly positive for lipase (C14), cystine arylamidase, *α*-chymotrypsin, *α*-galactosidase, *β*-glucosidase and *α*-mannosidase. The Biolog GEN III MicroPlate results showed that the strain was positive for dextrin, d-maltose, d-trehalose, d-cellobiose, gentiobiose, d-turanose, *α*-d-lactose, d-melibiose, *β*-methyl-d-glucoside, d-salicin, *N*-acetyl-d-glucosamine, *α*-d-glucose, d-mannose, d-galactose, l-rhamnose, d-galacturonic acid, l-galactonic acid lactone and d-glucuronic acid. The fatty acid composition of the strain includes summed feature 3 (C_16 : 1_* ω*7*c*/C_16 : 1_* ω*6*c*) as the main fatty acid, C_16 : 0_ and C_18 : 1_* ω*7*c*. The respiratory quinone of the strain is Q-8. The results of polar lipid analysis indicated DPG, PE, PG and PL. The genome has a G+C content of 47.82%. The type of the strain is NN19^T^ (=MCCC 1K08847^T^=KCTC 8393^T^). The genome and 16S rRNA gene sequences of the type strain are accession numbers JAYKKN000000000 and OR810597, respectively.

## Supplementary material

10.1099/ijsem.0.006827 Supplementary Material 1.

## Abbreviations

ANI: average nucleotide identity
BGCs: biosynthetic gene clusters
dDDH: digital DNA–DNA hybridization
DPG: diphosphatidylglycerol
KCTC: Korean Collection for Type Cultures
MCCC: Marine Culture Collection of China
MIS: Microbial Identification System
PE: phosphatidylethanolamine
PG: phosphatidylglycerol
PL: phospholipid
Q-8: ubiquinone-8
RAST: Rapid Annotation Subsystem Technology
TSA: tryptic soy agar
