# Supplementary material for: Cellvibrio chitinivorans sp. nov., a chitinolytic bacterium isolated from an intertidal mudflat
Source: Int J Syst Evol Microbiol. 2025 Jun 27;75(6):006827. doi: 10.1099/ijsem.0.006827 (PMC12281832; doi:10.1099/ijsem.0.006827)
Supplement: Supplementary Material 1. [file ijsem-75-06827-s001.pdf]

Supplementary Information

Cellvibrio chitinivorans **sp. nov., a chitinolytic bacterium isolated from an intertidal mudflat**

Supplementary Fig. S1. Transmission electron microscope of *Cellvibrio chitinivorans* NN19^T. Bar, 2.0 μm.

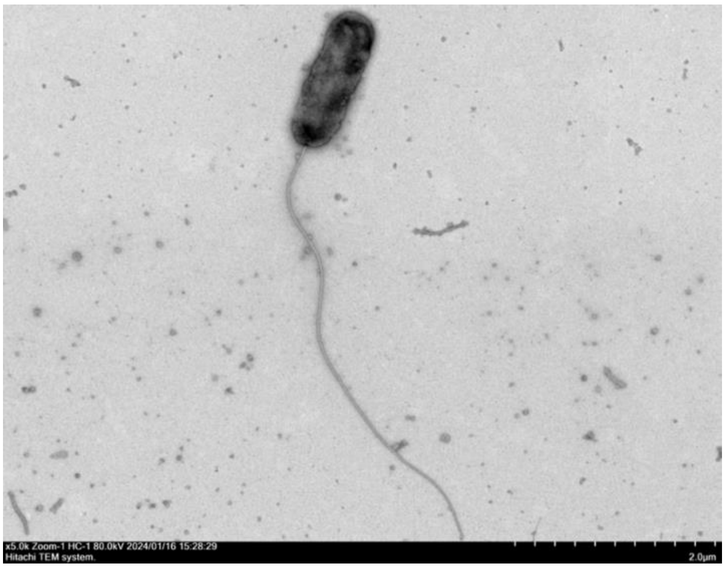

Supplementary Fig. S2. Polar lipids of strain NN19^T. PL, phospholipid; DPG, diphosphatidylglycerol; PE, phosphatidylethanolamine; PG, phosphatidylglycerol; F-first dimension of TLC; S- second dimension of TLC.

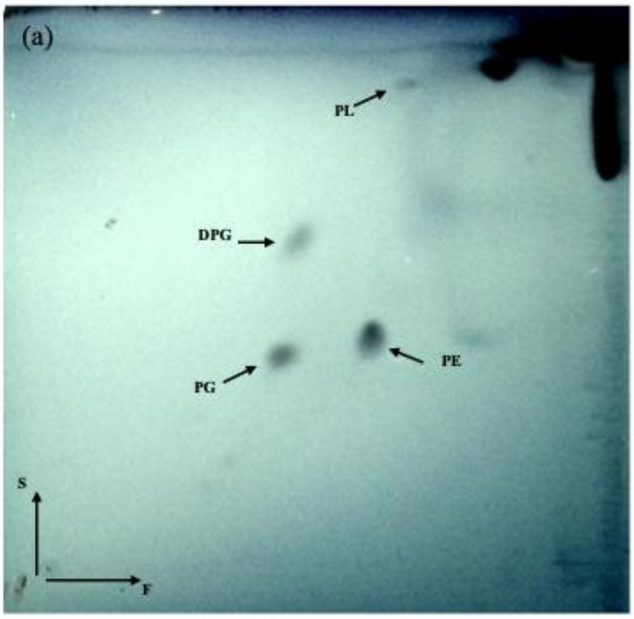

Supplementary Fig. S3. Minimum evolution tree showing the phylogenetic positions of strain NN19^T and related taxa based on 16S rRNA gene sequences. Bootstrap values (expressed as percentages of 1000 replications) are shown at branch points. Bar, 0.01 substitutions per nucleotide position.

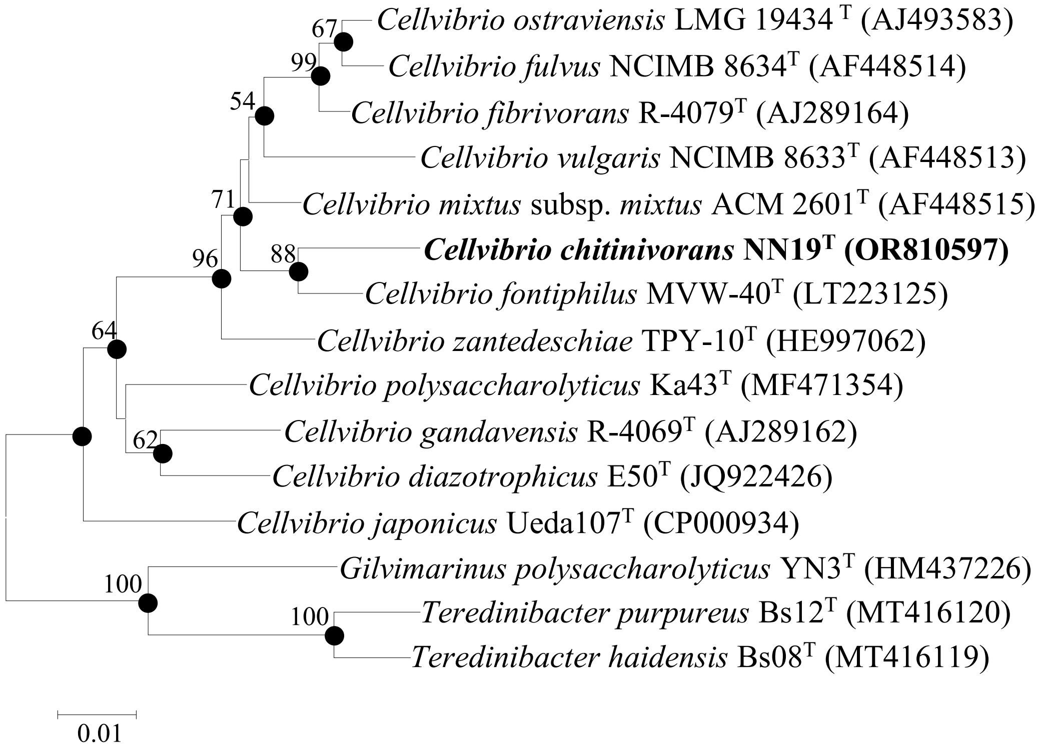

Supplementary Fig. S4. Neighbor-joining tree showing the phylogenetic positions of strain NN19^T and related taxa based on 16S rRNA gene sequences. Bootstrap values (expressed as percentages of 1000 replications) are shown at branch points. Bar, 0.01 substitutions per nucleotide position.

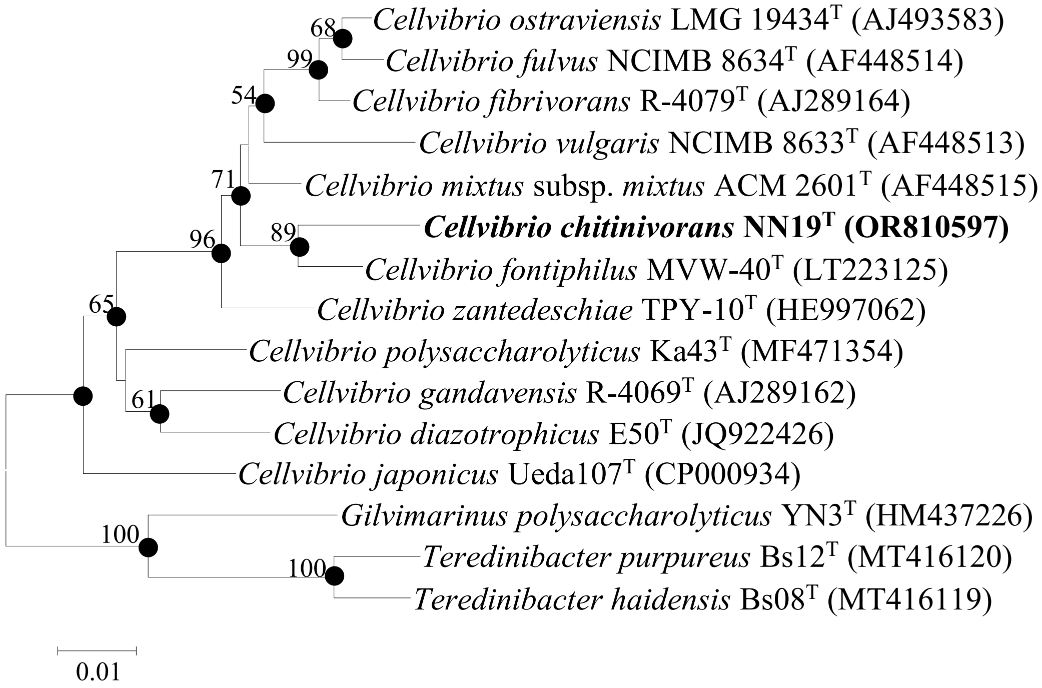

Supplementary Fig. S6. Results of 1% colloidal chitin degradation by strain NN19^T and its phylogenetically related strains after 48 h of incubation.

Strains: 1, uninoculated control group; 2, NN19^T; 3, *C. fontiphilus* KCTC 52237^T; 4, *C. mixtus* subsp. *mixtus* 21544^T.

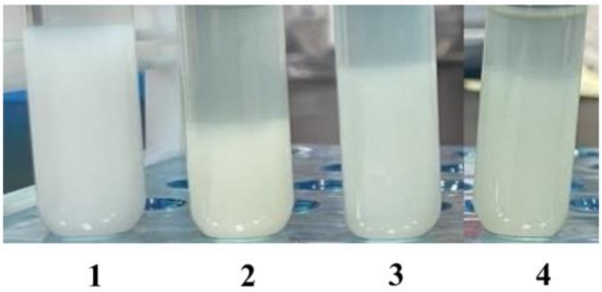

Supplementary Table S1. Comparative genomic analysis of strain NN19^T and closely related type strains based on RAST annotation results.

Strains: 1, NN19^T (JAYKKN000000000); 2, *C. fontiphilus* KCTC 52237^T (JAYKTU000000000); 3, *C. mixtus* subsp. *mixtus* J3-8^T (ALBT00000000); 4, *C. fibrivorans* BE90^T (JAVDVX000000000)

**characteristic**	**1**	**2**	**3**	**4**
Genome size (Mb)	4.4	4.1	5.2	5.1
G+C content (%)	47.8	49.5	46.5	48
Number of contigs	26	29	152	31
Protein-coding	3626	3424	4269	4176
Number of RNAs	47	45	43	45
**Subsystem Feature Counts:**				
Potassium metabolism	0	7	8	5
Virulence, Disease and Defense	38	35	37	44
Nitrogen Metabolism	15	10	10	11
Protein Metabolism	172	166	171	166
Stress Response	60	53	65	51
Sulfur Metabolism	11	8	18	8
Membrane Transport	117	99	122	116
Cofactors, Vitamins, Prosthetic Groups, Pigments	140	140	138	133
Dormancy and Sporulation	2	3	4	2
Carbohydrates	145	154	149	160

RNA Metabolism	50	48	50	53
Fatty Acids, Lipids, and Isoprenoids	37	39	39	36
Cell Wall and Capsule	30	23	25	25
Regulation and Cell signaling	32	30	25	30
Amino Acids and Derivatives	186	170	187	182
Phosphorus Metabolism	25	22	26	26
Iron acquisition and metabolism	2	6	0	3
Metabolism of Aromatic Compounds	14	17	13	14
DNA Metabolism	56	67	60	54
Nucleosides and Nucleotides	74	47	52	69
Phages, Prophages, Transposable elements, Plasmids	7	2	4	2
Motility and Chemotaxis	117	27	110	26
Respiration	58	56	92	59
Miscellaneous	9	8	22	18

Supplementary Table S2. The biosynthetic gene clusters (BGCs) of strain NN19^T and its closely related strains identified by antiSMASH.

Strains: 1, NN19^T (JAYKKN000000000); 2, *C. fontiphilus* KCTC 52237^T (JAYKTU000000000); 3, *C. mixtus* subsp. *mixtus* J3-8^T (ALBT00000000); 4, *C. fibrivorans* BE90^T (JAVDVX000000000)

**Strain**	Type	Size (kb)	Similarity Confidence	Most similar known cluster
**1**	ectoine	10	High	ectoine
	terpene-precursor	21	Low	indole
	arylpolyene	53	Low	APE Vf
	acyl_amino_acids	61	Low	pyoluteorin
	RiPP-like	11	-	-
**2**	ectoine	10	High	ectoine
	terpene-precursor	21	Low	indole
	NI-siderophore	21	Low	desferrioxamine E
	resorcinol	26	-	-
	arylpolyene	41	-	-
	betalactone	25	-	-
	hserlactone	21	-	-
	RiPP-like	11	-	-
**3**	arylpolyene	57	Low	APE Vf
	NRPS	29	Low	epaciachelin

	NI-siderophore	30	Low	alcaligin
	RiPP-like	11	-	-
	terpene-precursor	21	-	-
**4**	NI-siderophore	24	Low	alcaligin
	terpene-precursor	21	Low	indole
	arylpolyene	57	Low	APE Vf
	hserlactone	21	-	-
	betalactone	103	-	-
	T1PKS	46	-	-
	resorcinol	29	-	-

Supplementary Table S3. The Biolog GEN III assay results of NN19^T and its closely related strains. Strains: 1, NN19^T; 2, *C. fontiphilus* KCTC 52237^T; 3, *C. mixtus* subsp. *mixtus* LMG 21544^T. +, positive; −, negative; W, weakly positive; ND, not determined.

**Biolog GENIII assay**	**1**	**2**	**3**
Negative Control	-	-	-
Dextrin	+	+	+
D-Maltose	+	+	+
D-Trehalose	+	+	+
D-Cellobiose	+	+	+
Gentiobiose	+	+	+
Sucrose	W	+	+
D-Turanose	+	W	W
Stachyose	-	-	W
Positive Control	+	+	+
pH 6	+	+	+
pH 5	-	-	-
D-Raffinose	W	-	+
α-D-Lactose	+	+	+
D-Melibiose	+	+	+
β-Methyl-D-Glucoside	+	+	W
D-Salicin	+	+	+
N-Acetyl-D-Glucosamine	+	+	+
N-Acetyl-β-DMannosamine	-	-	-
N-Acetyl-D-Galactosamine	-	-	-
N-AcetylNeuraminic Acid	-	-	-
1% NaCl	+	+	+
4% NaCl	+	-	-
8% NaCl	-	-	-
α-D-Glucose	+	+	+
D-Mannose	+	+	+
D-Fructose	-	-	+
D-Galactose	+	+	+
3-Methyl Glucose	-	-	-
D-Fucose	-	-	-
L-Fucose	-	-	-
L-Rhamnose	+	-	+
Inosine	-	-	-
1% Sodium Lactate	W	W	W
Fusidic Acid	+	+	+
D-Serine	-	-	W
D-Sorbitol	-	-	-

D-Mannitol	-	-	-
D-Arabitol	-	-	-
myo-Inositol	-	-	-
Glycerol	-	-	-
D-Glucose-6-PO4	-	-	W
D-Fructose-6-PO4	-	-	W
D-Aspartic Acid	-	-	-
D-Serine	-	-	-
Troleandomycin	-	-	-
Rifamycin SV	+	+	-
Minocycline	-	-	-
Gelatin	-	-	-
Glycyl-L-Proline	-	-	-
L-Alanine	-	-	-
L-Arginine	-	-	-
L-Aspartic Acid	-	-	-
L-Glutamic Acid	-	-	-
L-Histidine	-	-	-
L-Pyroglutamic Acid	-	-	-
L-Serine	-	-	-
Lincomycin	+	+	+
Guanidine HCl	+	+	+
Niaproof 4	-	-	-
Pectin	-	+	+
D-Galacturonic Acid	+	+	+
L-Galactonic Acid Lactone	+	+	+
D-Gluconic Acid	-	-	-
D-Glucuronic Acid	+	+	+
Glucuronamid e	-	-	W
Mucic Acid	-	-	-
Quinic Acid	-	-	-
D-Saccharic Acid	-	-	-
Vancomycin	-	-	-
Tetrazolium Violet	-	W	-
Tetrazolium Blue	+	+	+
p-Hydroxy-Phenylacetic Acid	-	-	-
Methyl Pyruvate	-	-	-
D-Lactic Acid Methyl Ester	-	-	-
L-Lactic Acid	-	-	-
Citric Acid	-	-	-
α-Keto-Glutaric Acid	-	-	-
D-Malic Acid	-	-	-
L-Malic Acid	-	-	+
Bromo-Succinic Acid	-	-	W

Nalidixic Acid	-	-	-
Lithium Chloride	-	-	-
Potassium Tellurite	+	W	+
Tween 40	-	-	-
γ-Amino-ButryricAcid	-	-	-
α-Hydroxy-Butyric Acid	-	-	-
β-Hydroxy-D,LButyricAcid	-	-	-
α-Keto-Butyric Acid	-	-	-
Acetoacetic Acid	W	W	-
Propionic Acid	-	-	-
Acetic Acid	-	-	-
Formic Acid	-	-	-
Aztreonam	+	-	+
Sodium Butyrate	-	-	-
Sodium Bromate	-	-	-

Supplementary Table S4. The API ZYM assay results of NN19^{T} and its closely related strains. Strains: 1, NN19^{T}; 2, *C. fontiphilus* KCTC 52237^{T}; 3, *C. mixtus* subsp. *mixtus* LMG 21544^{T}. +, positive; −, negative; W, weakly positive; ND, not determined.

**API ZYM assay**	**1**	**2**	**3**
Water	-	-	-
Alkaline phosphatase	+	+	+
Esterase(C4)	+	+	+
Esterase lipase (C8)	+	+	+
Lipase (C14)	W	+	+
Leucine arylamidase	+	+	+
Valine arylamidase	+	+	+
Cystine arylamidase	W	+	W
Trypsin	-	+	W
α-chymotrypsin	W	+	-
Acid phosphatase	+	W	W
Naphtol-AS-BI-phosphohydrolase	W	W	W
α-galactosidase	W	+	+
β-galactosidase	-	W	W
β-glucuronidase	-	W	-
α-glucosidase	-	+	+
β-glucosidase	W	+	+
N-acetyl-β-glucosaminidase	+	+	+
α-mannosidase	W	W	W
β-fucosidase	-	W	-

Supplementary Table S5. The genome assembly results of strain NN19^T.

Assembly results	Scaffold	Contig
Total Number (>500bp)	25	26
Total Length (bp)	4,391,071	4,391,061
N50 Length (bp)	762,285	762,285
N90 Length (bp)	120,499	120,499
Max Length (bp)	1,573,620	1,573,620
Min Length (bp)	707	285
Sequence GC (%)	47.82	47.82

Supplementary Table S6. Genome completeness and contamination level statistics of strain NN19^T based on CheckM analysis.

Bin Id	NN19
Marker lineage	c__Gammaproteobacteria (UID4444)
# genomes	263
# markers	507
# marker sets	232
Completeness	100
Contamination	0.24
Strain heterogeneity	0
